# Advancing a Framework for Regulatory Use of Real-World Evidence: When Real Is Reliable

**DOI:** 10.1177/2168479018763591

**Published:** 2018-03-19

**Authors:** Nancy A. Dreyer

**Affiliations:** 1 IQVIA Real-World & Analytic Solutions, Cambridge, MA, USA

## Abstract

There is growing interest in regulatory use of randomized pragmatic trials and noninterventional real-world (RW) studies of effectiveness and safety, but there is no agreed-on framework for assessing when this type of evidence is sufficiently reliable. Rather than impose a clinical trial–like paradigm on RW evidence, like blinded treatments or complete, source-verified data, the framework for assessing the utility of RW evidence should be grounded in the context of specific study objectives, clinical events that are likely to be detected in routine care, and the extent to which systematic error (bias) is likely to impact effect estimation. Whether treatment is blinded should depend on how well the outcome can be measured objectively. Qualification of a data source should be based on (1) numbers of patients of interest available for study; (2) if “must-have” data are likely to be recorded, and if so, how and where; (3) the accessibility of systematic follow-up data for the time period of interest; and (4) the potential for systematic errors (bias) in data collection and the likely magnitude of any such bias. Accessible data may not be representative of an entire population, but still may provide reliable evidence about the experience of typical patients treated under conditions of conventional care. Similarly, RW data that falls short of optimal length of follow-up or study size may still be useful in terms of its ability to provide evidence for regulators for subgroups of special interest. Developing a framework to qualify RW evidence in the context of a particular study purpose and data asset will enable broader regulatory use of RW data for approval of new molecular entities and label changes. Reliable information about diverse populations and settings should also help us move closer to more affordable, effective health care.

## Background

The Commissioner of the US Food and Drug Administration (FDA), Dr Scott Gottlieb, has described the current process of drug development as unstable.^1^ In considering the high cost of drug development and new medicines, he recently posed this question: “Should a product be marketed based on a data set that speaks to a limited and rigidly constructed circumstance, when the clinical use, and in turn the evidence we might have to evaluate the product, could have been far richer, far more diverse, and more informative?”^2^ These rich, more diverse data come from real-world (RW) sources that were not created for research purposes but which, through appropriate study design and data curation, can be leveraged to understand cause-and-effect relationships between medical product use and outcomes. The Commissioner’s challenge—and the requirements of the 21st Century Cures Act—are generating attention and discussion about expanding regulatory uses of RW evidence beyond their more tradition use for evaluating product safety. The 21st Century Cures Act of 2016^3^ directs the FDA to evaluate the potential use of RW evidence to support approval of a new indication for an approved drug, and to support or satisfy postapproval study requirements (Section 3022). The FDA was given until December 2018 to develop a framework for these uses and an additional 3 years to issue guidance on these topics.

To flesh out the potential role of RWE, it is important to learn how evidence from both classical randomized clinical trial (RCT) and RW studies are currently being used by regulators. Classical RCTs are the most well-known and widely used approaches to evaluate the efficacy and safety of new medical products. Therapeutic benefits and risks are studied under strictly standardized conditions, in narrowly defined groups of people, and in situations that are most likely to show benefit, a construct that is reliable but expensive.^4^ However, the generalizability of the classical RCT to populations beyond the types of people studied is limited.^5^ Exploratory analyses of subgroup effects are frowned upon unless articulated at study outset and accounted for within a study that was designed with sufficient size to detect subgroup effects, should they exist. This practice is heavily influenced by a preoccupation with *P* values and the misperception that multiple statistical tests will lead to unjustified conclusions from distorted *P* values, regardless of the observed effect size.^6^

In fact, examination of subgroups that are not described in the initial protocol can reveal important information that identifies likely therapeutic responders as well as those at high risk of serious side effects. Consider lessons from the Systolic Blood Pressure Intervention Trial (SPRINT) sponsored by the National Institutes of Health that compared intensive management of systolic blood pressure (target <120 mm Hg) with standard management target (<140 mm Hg) among 9361 patients followed for 1 year.^7^ The trial was stopped early because intensive blood pressure management significantly lowered the rate of cardiovascular events, whether measured by composite endpoint and all-cause mortality. The journal editors later issued a challenge to use these trial data to identify a novel clinical finding that advances medical science.^8^ Subgroup analyses in patients with chronic kidney disease (CKD) showed a higher risk of therapy-related events from intensive blood pressure management, not a benefit.^9^ Without this invitation to look more deeply, more harm than good would have resulted from encouraging such intensive management in CKD patients.

RW evidence, which comes from using RW data, is a strong complement to RCTs because it provides the opportunity to systematically evaluate the use, benefits, and risks of medical products in more clinically diverse settings and patients, as well as under conditions that reflect the use of treatments in actual clinical practice. In contrast to the highly controlled way in which RCT data are generated, RW data comprise information about patients’ health status and/or the delivery of health care that generally may not have been collected for research purposes. RW evidence can be assembled from a variety of data sources, including data elements captured in a patient’s electronic health record (EHR), in a hospital or insurance company’s administrative and claims data, directly from patents or providers in the course of an observational study, from sources of patient-generated information outside of clinical settings (eg, in-home monitors and wearables), and in registries that support various aspects of care and research.^10^

Recognizing that both RCT and RW evidence have important roles in shaping understanding of clinical risks and benefits, developing a fit-for-purpose framework for evaluating the validity of RW evidence will allow its use more broadly by regulators, allowing RW data to take a proper place alongside those from RCTs. The task at hand centers around developing agreement on what constitutes good practice and appropriate use.^11^

Regulators would benefit from a framework in which to evaluate RW evidence in the context of specific medical products and conditions to determine if and when RW studies are reliable enough for meaningful interpretation. Specifically, a closer look is needed at which methodological and procedural aspects are essential for regulatory decision making and which aspects may be desirable but not essential for every indication. Key applications of the framework should include the use of RW data comparators with single-arm trials and registries, as well as describing the key elements required in randomized pragmatic trials. Recent examples of regulatory use of RW data are presented, along with a description of 4 core elements for a framework to evaluate RW evidence framework and the rationale for their inclusion.

### Understanding Current Regulatory Use of RW Evidence

In what situations have regulators accepted RW evidence for product approval or label expansion (observational studies and RW data used as comparators for single arm clinical trials)?

#### Approval of new molecular entities and label expansions for rare diseases

Currently regulators do not require randomized trials in situations where it would be unethical or infeasible, such as in studies of treatments for rare diseases in which no adequate treatment exists. RW data in the form of historical controls have been used to support successful regulatory applications for nearly 50 years. Now, rather than looking back in time for the purpose of comparison, RW data are being used to provide more contemporaneous benchmarks. Consider the recent approval of avelumab (a programmed death ligand [PD-L1] blocking human IgG1 lambda monoclonal antibody) as monotherapy for metastatic Merkel cell carcinoma (mMCC) in the US^12^ and Europe.^13^ RW data were used to characterize the natural history of mMCC and were offered to regulators as benchmarks, not a formal comparator arm. A subset of trial patients who responded well to treatment was identified and the benefit documented through contrast with the RW benchmarks data, leading to regulatory approval of this new molecular entity in the US, European Union, and Japan for that subset.^14,15^

The avelumab story is not an isolated example. RW data comparators were also used to support a conditional authorization from the European Medicines Agency (EMA) for a new advanced therapy medicine product, a cell-based treatment called Zalmoxis, for a rare disease. In this case, adults received a haploidentical, hematopoietic stem cell transplant for various types of blood cancer to aid immune reconstitution and reduce the risk of graft versus host disease.^16^ Here the treatment had a clear endpoint that was shown to be successful in a phase 1/2 trial, and data from a European Transplant registry was used to provide comparison data about how similar patients fared without the treatment.

RW data are also used for label expansions in situations where information about treatment effectiveness and safety becomes available through continued patient study outside of the clinical trial paradigm, as was the case with the recent label expansion granted by the EMA for eculizumab. Data from an observational, international disease registry were used to support a label expansion in paroxysmal nocturnal hemoglobinuria, an ultra-rare, life-threatening disease of the blood. The original labeling requiring patients to have had a transfusion before using eculizumab was eliminated based on real-world data that showed a clinical benefit in patients with elevated hemolysis and the presence of related clinical symptoms, regardless of transfusion history.^17^

#### Label expansions for common conditions

Natural history comparators based on RW data, also called synthetic comparators, continue to be important tools for regulators even outside of the rare disease paradigm. For example, the FDA has agreed to accept RW data as a comparator arm for a label expansion for a class III medical device that was first approved in 1994. Here we see modern epidemiologic methods being used to evaluate the outcome of interest by comparing the experience of patients participating in a device registry with matched RW comparators. By way of background, the EXOGEN device, an ultrasound bone healing system, was originally approved for fracture healing in established non-unions (excluding skull and vertebrae) and conservatively treated fresh fractures of the tibia and radius. Since its approval, the device has been more broadly used and is reimbursed off-label by most commercial insurers. A formal study of its effectiveness in other bones except skull/vertebrae is being conducted for the purposes of regulatory submission. Data from a registry of patients treated with the EXOGEN device will be compared to propensity score–matched patients from health insurance claims data.^18^ In contrast to a classical RCT design, people treated with EXOGEN are contacted periodically, using a direct-to-patient approach to understand if the fracture has healed. Patient-reported outcomes will be validated through the presence of a non-union diagnosis in the medical billing records of treating clinicians. External comparators will be obtained from health insurance claims that capture medical history, in which the outcome of nonunion is identifiable by the presence of that diagnosis in the claims data.

### Crafting a Framework for Broader Use of RW Evidence

#### Is randomized treatment assignment needed?

Although there are robust design and analytic approaches that have been developed for noninterventional studies of comparative effectiveness and safety,^19^ they are not yet widely understood and they involve acceptance of some assumptions and uncertainties. In contrast, randomization is a well-respected method used to balance patient characteristics between treatment arms, and is an accepted, important characteristic of an “adequate” and “well controlled” clinical trial intended for regulatory submission.^20^ Randomized can also be used in pragmatic trials, which are increasingly being conducted in a variety of settings including (1) at the point of routine care; (2) within registries, which allows for systematic data collection after randomization (sometimes referred to as a randomized registry trial); and (3) embedded in health systems where EHR and health insurance claims data are used as sources of follow-up data.^21–23^ Randomized treatment assignments eliminate the channeling bias seen in noninterventional studies where, in everyday clinical practice, treatments are chosen because of individual patient characteristics including severity of the condition and the perceived likelihood that a patient can afford, will accept and use the recommended treatment(s). In RW studies, randomization is also favored when a treatment is newly approved and payers have not yet approved reimbursement. However, it is essential that randomization only be used in situations of clinical equipoise. When there is genuine uncertainly about the choice between 2 or more care options, randomized pragmatic trials can be excellent tools to provide clinical evidence that would influence future care.

That said, randomization is not always feasible or ethical, as in examples of rare diseases, or in situations where sham treatments introduce unacceptable patient risk, such as with certain types of surgeries.

#### Must treatment be blinded?

The value of blinded treatment, another basic tenet of classical RCTs, is that it protects against biased interpretation due to known exposures, so that knowledge of which treatment was used will not influence an investigator’s assessment of benefit or harm. For some uses, the scientific benefits of blinded treatments may be outweighed by practical implications for site and patient participation in trials in terms of cost, logistics, and investigator requirements. Providing blinded treatments requires working with investigators who can appropriately store, distribute, and account for their use, which makes it difficult to include a broad spectrum of investigators and sites. Blinding also imposes artificial constraints on patient behavior since patients must obtain all their treatments through the trial investigators instead of using retail outlets or arranging for treatments that are administered in hospital or outpatient settings. Patients are unlikely to tell the study investigator when they are not using the treatments as intended—either taking them less frequently, at inappropriate times, or not at all. They may continue to participate in trials because of the benefits of paid-for special medical care or consultation with specialists not available to them outside of the clinical trial setting. Additionally, treatment adherence may not reflect RW situations since no payment or co-payment for treatment or medication is required.

- When blinding is needed: Blinding is particularly useful for outcomes that cannot be measured objectively, such as quality of life, pain, or depression. Consider a recent study that assessed volunteers’ response to anti-itch medications in an experiment where 2 inactive treatments were disguised so that one appeared to be an expensive-looking, brand-name product and the other, a generic.^24^ Patients were told that the cream could increase sensitivity to pain and then were randomized to treatment and exposed to heat. Patients who had been randomized to the expensive-appearing treatment consistently experienced greater sensitivity to pain, confirmed by MRI, than those who thought they had been treated with an inexpensive cream, showing how much perception influenced response. That said, it is worth considering when blinding is critical for a study outcome since the costs related to supervision, distribution, and accounting for blinded drug use can account for as much as roughly one-third of the budget for a classical RCT.
- When blinding is less useful: There are many outcomes for which a patient or clinician’s knowledge about treatment will not substantially affect event reporting. Outcomes based on objective tests and measurements that are performed in everyday situations, such as tumor size, white blood cell count, and death, should be considered fairly reliable and not subject to observer bias. In addition, there may be data that are collected systematically that closely approximate outcomes of interest. For example, many of the major adverse cardiac events (MACE) that are evaluated as part of a regulatory commitment for new antidiabetics^25^ are conditions that can be objectively assessed regardless of rater knowledge, such as death or hospital admission for a presumed cardiac event.

A recent test of this concept was performed by comparing cardiovascular outcomes that had been adjudicated by study physicians in the context of an RCT with Medicare claims data for those same patients.^26^ Data for patients who were continuously enrolled in traditional fee-for-service Medicare Part A coverage were used to identify myocardial infarctions (primary outcome) and coronary revascularization (secondary outcome). There was good agreement between adjudicated outcomes for MI and claims (RW) data, and even strong agreement for coronary revascularization. More important, when analyses of the original RCT data were compared with analysis based purely on claims data, the effect estimates for myocardial infarction were nearly identical (hazard ratios of 1.31 with 95% confidence interval [CI] of 1.03-1.67 from trial data compared with 1.29, 95% CI of 1.00-1.35, from claims data.) The 2 approaches also matched equally closely for coronary revascularization (1.09, 95%CI 0.88-1.35, for trial data and 1.10, 95% CI 0.89-1.35, from claims data). This example illustrates the benefit of using RW data for these cardiovascular outcomes.

#### What are the “pragmatic” trade-offs compared to classical RCTs?

Both classical RCTs and randomized pragmatic trials adhere to the principles of Good Clinical Practice. However, unlike classical RCTs,

- Pragmatic trials never use placebos, instead comparing a treatment of interest to one or more active treatments in use in the region.
- Surrogate measures are rarely used in pragmatic trials. Instead they are generally designed to study clinical events that come to attention through ordinary medical care in the region under study. The outcomes have meaning to clinicians and patients, such as clinical endpoints that are direct measures of how a patient feels, functions, or survives, including resolution or prevention of symptoms.^27^ Patients are typically followed during the ordinary course of care, their treatment use recorded and the outcomes analyzed in relation to treatment.

A big distinction between RCTs and pragmatic trials centers around the trade-offs between internal and external validity. RCTs have strong internal validity, achieved in large part through onsite study monitoring, but limited generalizability. In contrast, pragmatic trials generally focus on strengthening external validity by including more diverse types of patients, medical care providers, and settings, and often observing patients over longer time periods than most classical RCTs, for example, often several years for RW evidence in contrast to 3 to 4 months for many RCTs. However, pragmatic trials have less internal validity than RCTs. Site and data monitoring is rarely performed and, if done, is often conducted remotely. If monitoring is performed, source data verification (SDV) on the order of 20% is accepted as high quality, using the benchmark for higher-quality patient registries.^28^

Not surprisingly, a key difference between pragmatic trials and classical RCTs is cost. Two of the major cost drivers for RCTs relate to the use of blinded treatments and 100% SDV, in addition to costs for provisioning drug and comparators (placebo or active comparator), as needed. The major savings in the pragmatic trial comes from using open label treatments with treatment choices that would be encountered in real-world settings (“standard of care” comparators), and from selective site monitoring with greater attention to those sites that appear to be performing poorly. Consider this example: a phase 3b cardiovascular outcomes trial (CVOT) intended for use as a label expansion, based on the assumption that a study size of 5000 patients would be needed to detect 844 events over 90 days. The outcomes of interest are the MACE typical to CVOTs. The various features and cost of a classical RCT are compared with a randomized pragmatic trial in Table 1. The randomized pragmatic trial is substantially less costly than the classical RCT in this example, and is likely to generate evidence reliable enough for regulatory decision support since MACE are generally evident in RW settings.

**Table 1.** Contrasting a Classical RCT and a Randomized Pragmatic Trial for Cardiovascular Outcomes Trial.

	Study Type	
	Classical Phase 3b Randomized Clinical Trial	Randomized Pragmatic Trial
Study specifications		
5,000 patients 24 countries 587 sites	Randomized treatment Double blinded Placebo-controlled	Randomized treatment Open label Standard of Care comparators
Outcome: Major Adverse Cardiovascular Events (MACE)	Classical MACE definition Study physicians blinded to treatment and blinded adjudication	MACE slightly modified for RW Study physicians are aware of treatments, but blinded endpoint adjudication
Monitoring and Source Data Verification (SDV)	Full monitoring/ SDV	Risk based, remote monitoring
Total cost of study excluding drug and comparators	$110 M	$40 M
Cost	$22,000/patient	$8,000/patient

Abbreviations: M, million; RCT, randomized clinical trial; RW, real world.

### Recommendations for a Framework for Regulatory Use of RW Evidence

The idea of RW evidence to supplement RCTs has great allure as a faster and often lower-cost solution to generating evidence. However, its successful use requires a paradigm shift for drug developers. First, the clinical outcomes must be practical, patient-centric, and likely to be detected and recorded under conditions of everyday medical care. Second, the data source must be fit for purpose in terms of its utility for a particular clinical question of interest, target population, and length of follow-up.

The utility of RW data can generally be assessed by determining the following:

1. If sufficient numbers of patients of interest are likely to be available for a study after applying the patient inclusion and exclusion criteria
2. How well available data characterizes the exposures and outcomes of interest, recognizing that information recorded in structured fields are easier to find and analyze than unstructured notes, which may not even be accessible to researchers
3. How likely it is that patients will be followed in the data source for the desired length of follow-up
4. The potential for systematic error (bias) in data collection, and the likely magnitude of any such bias

A good understanding about the health system from which the data are derived will shed light on the limitations of any data source. For example, different products and services are captured according to health care coverage and health system; for example, private insurance systems may provide different coverage than regional or national systems. Items and events that are not covered by a health system or health care insurer will not be recorded in those systems, such as treatments that are not reimbursed. There also may be age or employment restrictions that affect the characteristics of people for which information is available. If hospitalizations are important outcomes, it is important to recognize that ambulatory care EHR data may be missing information completely about such events or may only note the fact of hospitalization but no detailed information about the care provided. Also important, patients may receive care from a different institution after diagnosis, such as patients who go to a tertiary care center for diagnosis and treatment advice but who may then obtain follow-up care from clinicians closer to their homes, in which case information on actual treatment and outcomes may not be accessible from the tertiary care center records. Thus, it is important to understand who is recording the information, where and why, data flow, coding and classification systems as well as the processes used for data curation, validation, standardization and transfer.

Even though some potential for systematic error may be discovered, it is important to put that likelihood in context. For example, missing outcome data for a substantial portion of the study population would be quite problematic. In contrast, recognizing that not all patients return to a health system for care may be less problematic, unless there is good evidence to show selective loss to follow-up for people who have better or worse outcomes than those who stay in the health system.

Privacy and data access rules, ethical approval requirements, contracting, governance, and funding (as a gauge of sustainability) must be considered. It is also useful to understand which components of the database are sensitive to change, and how to further evaluate and rectify such issues in analyses.^29^ In addition, it is important to document if and how natural language processing and/or artificial intelligence were used to assist in characterizing large and diverse data. If data elements are to be combined from different data sources, a common data model may be used and if so, it is desirable to know how it is supported and what tools have been built for its application. Using a common data model makes it easier to integrate data across health systems, but post hoc common data models can also be applied.

## Conclusion

Although classical RCTs have been the historical standard for obtaining unbiased information about the benefits and risks of new drugs and for label expansions, there is growing use of RW evidence by regulators to support broader use of effective therapies, and a recognition that RW data, even in the absence of randomization, can contributed useful information about treatment effectiveness.^30,31^ Furthermore, using RW data that falls short of the optimal length of follow-up or study size may still be useful in terms of its ability to provide useful evidence for regulators relating to subgroups of special interest, as was demonstrated in the avelumab example.^32^ However, just because RW data exist does not mean that those data will be useful for every research question.

To facilitate use of RW evidence to support regulatory filings sufficient information should be provided to facilitate description and evaluation of the RW data and methods used, as well as any known limitations. The data source should be described including the selected data fields, how those fields have been populated, for what purpose the information was originally collected by the source, whether the information was provided by the source in native form or had been modified prior to delivery, whether fields are structured or unstructured, and parameters limiting or shaping content that populates fields. Methodologies employed to modify the original data either by the source or the recipient should also be described, including how derived variables were created.

In summary, the key lessons for using RW evidence for regulatory purposes are to 1) qualify RW data sources in the context of specific study objectives and 2) apply intelligent designs using outcome measures based on RW practices. The outcomes measured, circumstances under which the data were recorded and populations covered should be placed in the context of other evidence and treatment options. The framework for assessing RW data quality must be grounded in the context of specific study objectives that are measurable in actual clinical practice. An agreed-upon framework will facilitate use of study-specific RW evidence as well as evidence derived from platforms that support repeated uses as follow-up accumulates and new questions arise. A disciplined, structured approach tailored to RW evidence should enhance the ability of regulators to critically examine both RCT and RW evidence to approve safe and effective medical products more quickly and possibly, even more affordably.
